# New 9-Hydroxybriarane Diterpenoids from a Gorgonian Coral *Briareum* sp. (Briareidae)

**DOI:** 10.3390/ijms17010079

**Published:** 2016-01-09

**Authors:** Yin-Di Su, Chun-Sung Sung, Zhi-Hong Wen, Yu-Hsin Chen, Yu-Chia Chang, Jih-Jung Chen, Lee-Shing Fang, Yang-Chang Wu, Jyh-Horng Sheu, Ping-Jyun Sung

**Affiliations:** 1 Department of Marine Biotechnology & Resources and Asia-Pacific Ocean Research Center, National Sun Yat-sen University, Kaohsiung 804, Taiwan; gobetter04@yahoo.com.tw (Y.-D.S.); wzh@mail.nsysu.edu.tw (Z.-H.W.); 2 National Museum of Marine Biology & Aquarium, Pingtung 944, Taiwan; kb5634@yahoo.com.tw (Y.-H.C.); jay0404@gmail.com (Y.-C.C.); 3 Department of Anesthesiology, Taipei Veterans General Hospital, Taipei 112, Taiwan; sung6119@gmail.com; 4 School of Medicine, National Yang-Ming University, Taipei 112, Taiwan; 5 Doctoral Degree Program of Marine Biotechnology, National Sun Yat-sen University & Academia Sinica, Kaohsiung 804, Taiwan; 6 Department of Life Science and Institute of Biotechnology, National Dong Hwa University, Hualien 974, Taiwan; 7 Department of Pharmacy & Graduate Institute of Pharmaceutical Technology, Tajen University, Pingtung 907, Taiwan; jjchen@tajen.edu.tw; 8 Department of Sport, Health and Leisure, Cheng Shiu University, Kaohsiung 833, Taiwan; lsfang@csu.edu.tw; 9 School of Pharmacy, College of Pharmacy, China Medical University, Taichung 404, Taiwan; 10 Chinese Medicine Research and Development Center, China Medical University Hospital, Taichung 404, Taiwan; 11 Center for Molecular Medicine, China Medical University Hospital, Taichung 404, Taiwan; 12 Graduate Institute of Natural Products, Kaohsiung Medical University, Kaohsiung 807, Taiwan; 13 Graduate Institute of Marine Biology, National Dong Hwa University, Pingtung 944, Taiwan

**Keywords:** *Briareum*, briarenolide, briarane, gorgonian, anti-inflammatory, iNOS

## Abstract

Six new 9-hydroxybriarane diterpenoids, briarenolides ZI–ZVI (**1**–**6**), were isolated from a gorgonian coral *Briareum* sp. The structures of briaranes **1**–**6** were elucidated by spectroscopic methods and by comparison of their spectroscopic data with those of related analogues. Briarenolides ZII (**2**) and ZVI (**6**) were found to significantly inhibit the expression of the pro-inflammatory inducible nitric oxide synthase (iNOS) protein of lipopolysaccharide (LPS)-stimulated RAW264.7 macrophage cells.

## 1. Introduction

**Figure 1:**
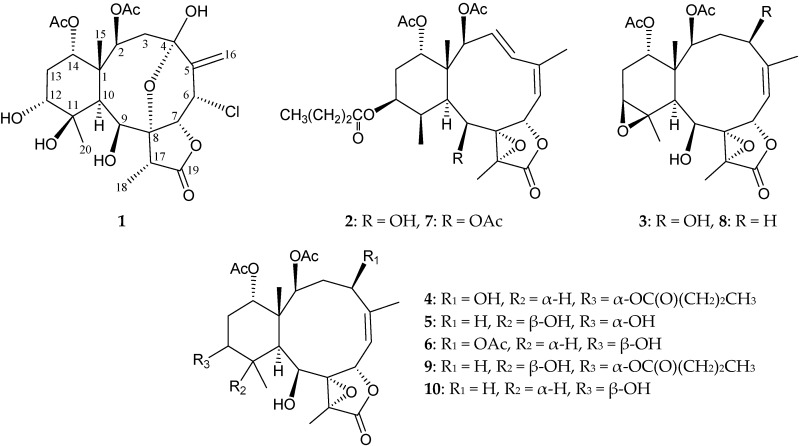
The structures of briarenolides ZI–ZVI (**1**–**6**), excavatolide F (**7**), 2β-acetoxy-2- (debutyryloxy)-stecholide E (**8**), excavatolide Z (**9**) and excavatolide E (**10**).

The briarane-type diterpenoid (3,8-cyclized cembranoid), 2β-acetoxy-2-(debutyryloxy)-stecholide E, was first isolated from the gorgonian coral *Briareum* sp. in 1996 [1]. Since then, hundreds of compounds of this type have been obtained from various Taiwanese gorgonian corals, such as *Briareum*, *Junceella* and *Ellisella* spp. [2,3,4,5,6], that have been located off the coast of Taiwan. Recently, in a sample collected at the southern tip of Taiwan, as *Briareum* sp. (family Briareidae), we identified six new briaranes, briarenolides ZI–ZVI (**1**–**6**) (Figure 1). In this report, we isolate and determine the structures of these briaranes, in addition to studying their anti-inflammatory properties.

## 2. Results and Discussion

The molecular formula of a new briarane, briarenolide ZI (**1**), was determined as C_24_H_33_ClO_11_ (eight degrees of unsaturation) by high-resolution electrospray ionization mass spectrum (HRESIMS) at *m*/*z* 555.16025 (calcd. for C_24_H_33_ClO_11_ + Na, 555.16036). The IR of **1** showed absorptions at 1715, 1769 and 3382 cm^−1^, which were consistent with the presence of ester, γ-lactone and hydroxy groups. The ^13^C NMR spectrum (Table 1) suggested that **1** possessed an exocyclic carbon-carbon double bond based on signals at δ_C_ 138.6 (C-5) and 116.9 (CH_2_-16), which was confirmed by the ^1^H NMR spectrum of **1** (Table 1), which showed two olefin proton signals at δ_H_ 5.88 (1H, dd, *J* = 2.4, 1.2 Hz, H-16a) and 5.64 (1H, dd, *J* = 2.4, 1.2 Hz, H-16b). Three carbonyl resonances at δ_C_ 175.3 (C-19), 173.4 and 169.3 (2 × ester carbonyls) revealed the presence of one γ-lactone and two ester groups in **1**; two acetyl methyls (δ_H_ 2.06, s, 2 × 3H) were also observed. According to the overall unsaturation data, it was concluded that **1** was a diterpenoid molecule possessing four rings.

^1^H NMR coupling information in the ^1^H–^1^H correlation spectroscopy (COSY) spectrum of **1** enabled identification of the H-2/H_2_-3, H-6/H-7, H-12/H_2_-13/H-14, H-6/H_2_-16 (by allylic coupling) and H-17/H_3_-18 units (Table 1). The heteronuclear multiple bond coherence (HMBC) correlations between protons and quaternary carbons of **1** (H-2, H_2_-3, H-10, H_2_-13, H_3_-15/C-1; H-2, H_2_-3, H_2_-16, OH-4/C-4; H-16b, OH-4/C-5; H-10, H_3_-18, OH-9/C-8; H_3_-20/C-11 and H-17, H_3_-18/C-19) permitted elucidation of the carbon skeleton (Table 1). HMBC correlations between H_2_-16/C-4, -5 and -6 indicated an exocyclic double bond at C-5, which was further confirmed by the allylic coupling between H_2_-16/H-6. HMBC correlations between H_3_-15/C-1, -2, -10 and -14 and H-2 and H-10/C-15, revealed that the ring junction C-15 methyl group was located at C-1. Furthermore, an HMBC correlation between H-2 (δ_H_ 5.09) and the acetate carbonyl (δ_C_ 173.4) revealed the presence of an acetate ester at C-2; and an HMBC correlation between a hydroxy proton (δ_H_ 6.50) and C-4 oxygenated quaternary carbon suggested the presence of a hydroxy group at C-4. The C-4 hydroxy group was determined to be part of a hemiketal constellation on the basis of a characteristic carbon signal at δ_C_ 96.7. ^1^H–^1^H COSY correlations between OH-9/H-9 and OH-12/H-12 suggested the presence of the hydroxy groups at C-9 and C-12. A carbon signal at δ_C_ 81.8 (C-8) indicated ^3^*J*-coupling with protons at δ_H_ 2.23 (H-10), 1.33 (H_3_-18) and 2.73 (OH-9). Therefore, the remaining hydroxy and acetoxy groups had to be positioned at C-11 and C-14, respectively, as indicated by analysis of ^1^H–^1^H COSY correlations and characteristic NMR signal analysis. The intensity of the sodiated molecules [M + 2 + Na]^+^ isotope peak observed in the ESIMS and HRESIMS spectra ([M + Na]^+^:[M + 2 + Na]^+^ = 3:1) was evidence of the presence of one chlorine atom in **1**. The methine unit at δ_C_ 56.2 was more shielded than expected for an oxygenated carbon and was correlated to the methine proton at δ_H_ 5.54 (H-6) in the heteronuclear multiple quantum coherence (HMQC) spectrum, and this proton signal was ^3^*J*-correlated with H-7 (δ_H_ 4.73) in the ^1^H–^1^H COSY spectrum, which proved that a chlorine atom was attached at C-6. These data, together with the HMBC correlations between H-17/C-9, -18 and -19 and H_3_-18/C-8, -17 and -19, established the molecular framework of **1**.

**Table 1:** ^1^H (400 MHz, CDCl_3_) and ^13^C (100 MHz, CDCl_3_) NMR data and ^1^H–^1^H COSY (correlation spectroscopy) and HMBC (heteronuclear multiple bond coherence) correlations for briarane **1**.

Position	δ_H_ (*J* in Hz)	δ_C_, Multiple	^1^H–^1^H COSY	HMBC
1	–	45.6, C	–	–
2	5.09 d (6.4)	73.4, CH	H_2_-3	C-1, -4, -15, acetate carbonyl
3	3.73 dd (16.0, 6.4); 1.46 d (16.0)	41.7, CH_2_	H-2	C-1, -2, -4
4	–	96.7, C	–	–
5	–	138.6, C	–	–
6	5.54 dt (2.8, 2.4)	56.2, CH	H-7, H_2_-16	n. o. ^a^
7	4.73 d (2.8)	79.8, CH	H-6	n. o.
8	–	81.8, C	–	–
9	4.88 d (3.2)	76.9, CH	H-10, OH-9	n. o.
10	2.23 s	40.5, CH	H-9	C-1, -2, -8, -9, -15
11	–	78.5, C	–	–
12	3.50 br s	76.1, CH	H_2_-13, OH-12	n. o.
13	2.44 ddd (15.6, 4.0, 2.8); 1.98 ddd (15.6, 3.2, 2.8)	28.0, CH_2_	H-12, H-14	C-1
14	5.22 t (2.8)	76.3, CH	H_2_-13	n. o.
15	1.55 s	16.5, CH_3_	–	C-1, -2, -10, -14
16a/b	5.88 dd (2.4, 1.2); 5.64 dd (2.4, 1.2)	116.9, CH_2_	H-6	C-4, -5, -6
17	2.58 q (7.2)	50.4, CH	H_3_-18	C-9, -18, -19
18	1.33 d (7.2)	8.2, CH_3_	H-17	C-8, -17, -19
19	–	175.3, C	–	–
20	1.56 s	28.9, CH_3_	–	C-10, -11, -12
OAc-2	–	173.4, C	–	–
	2.06 s	21.3, CH_3_	–	Acetate carbonyl
OAc-14	–	169.3, C	–	–
	2.06 s	21.1, CH_3_	–	Acetate carbonyl
OH-4	6.50 s	–	–	C-3, -4, -5
OH-9	2.73 d (3.2)	–	H-9	C-8
OH-12	2.67 br s	–	H-12	n. o.

^a^ n. o. = not observed.

**Figure 2:**
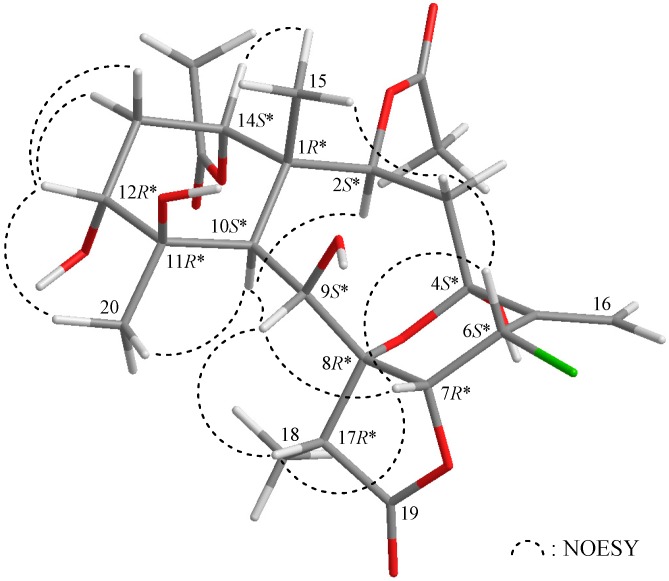
Selected protons with key nuclear Overhauser effect spectroscopy (NOESY) correlations of **1**.

The relative configuration of **1** was elucidated on the basis of a nuclear Overhauser effect spectroscopy (NOESY) experiment and by vicinal ^1^H–^1^H proton coupling constant analysis. Most naturally-occurring briarane natural products have Me-15 in the β-orientation and H-10 in the α-orientation [2,3,4,5,6], which were verified by the absence of a correlation between these two groups. In the NOESY experiment of **1** (Figure 2), H-10 correlated with H-2, H-9 and H_3_-20, indicating that these protons were situated on the same face; they were assigned as α protons, as C-15 methyl was β-oriented at C-1. The oxymethine proton H-14 was found to exhibit a response with H_3_-15, but not with H-10, revealing that H-14 was β-oriented. H-12 correlated with each of the C-13 methylene protons and H_3_-20, but not with H-10, indicating that H-12 was β-oriented and was positioned on the equatorial direction in the cyclohexane ring by modeling analysis. H-17 exhibited correlations with H-9 and H-7 and was also found to be reasonably close to H-9 and H-7 by modeling analysis; thus, H-17 could therefore be placed on the β face in **1**, and H-7 was β-oriented. One of the C-3 methylene protons (δ_H_ 3.73) displayed a correlation with H_3_-15; therefore, it was assigned as the H-3β proton, and the other was assigned as H-3α (δ_H_ 1.46). H-6 displayed correlations with H-3β and H-7, which confirmed that this proton was in the β-orientation, and the oxygen bridge between C-4 and C-8 was found to be α-oriented by modeling analysis. Based on the aforementioned results, the structure, including the relative configuration, of **1** was elucidated unambiguously.

Briarenolide ZII (**2**) was isolated as a white powder and had a molecular formula of C_28_H_38_O_10_ on the basis of HRESIMS at *m*/*z* 557.23552 (calcd. for C_28_H_38_O_10_ + Na, 557.23572). Carbonyl resonances in the ^13^C NMR spectrum of **2** (Table 2) at δ_C_ 173.0, 170.7, 170.4 and 169.9 demonstrated the presence of a γ-lactone and three other esters in **2**. It was found that the NMR signals of **2** were similar to those of a known briarane analogue, excavatolide F (**7**) [7] (Figure 1), except that the signals corresponding to the 9-acetoxy group in **7** were replaced by signals for a hydroxy group in **2**. The correlations from a NOESY experiment of **2** also revealed that the stereochemistry of this metabolite was identical to that of **7**. Thus, briarenolide ZII (**2**) was found to be the 9-*O*-deacetyl derivative of **7**.

Briarenolide ZIII (**3**) had a molecular formula C_24_H_32_O_10_ as deduced from HRESIMS at *m*/*z* 503.18858 (calcd. for C_24_H_32_O_10_ + Na, 503.18877). The IR spectrum of **1** showed three bands at 3444, 1779 and 1732 cm^−1^, which were in agreement with the presence of hydroxy, γ-lactone and ester groups. Carbonyl resonances in the ^13^C NMR spectrum of **3** at δ_C_ 171.8, 170.7 and 170.6 revealed the presence of a γ-lactone and two esters (Table 3). Both esters were identified as acetates by the presence of two acetyl methyl resonances in the ^1^H (δ_H_ 2.01, 1.98, each 3H × s) and ^13^C (δ_C_ 21.1, 21.1) NMR spectra (Table 3).

It was found that the NMR data of **3** were similar to those of a known briarane analogue, 2β-acetoxy-2-(debutyryloxy)-stecholide E (**8**) [1] (Figure 1), except that the signals corresponding to the 4-hydroxy group in **3** were not present in **8**. A correlation from the NOESY signals of **3** showed that H-4 correlated with H-2, but not with H_3_-15, indicating that the hydroxy group at C-4 was β-oriented. The results of ^1^H–^1^H COSY and HMBC correlations fully supported the positions of functional groups, and hence, briarenolide ZIII (**3**) was found to be the 4β-hydroxy derivative of **8**.

**Table 2:** ^1^H (400 MHz, CDCl_3_) and ^13^C (100 MHz, CDCl_3_) NMR data and ^1^H–^1^H COSY and HMBC correlations for briarane **2**.

Position	δ_H_ (*J* in Hz)	δ_C_, Multiple	^1^H–^1^H COSY	HMBC
1	–	45.6, C	–	–
2	5.39 d (10.0)	75.9, CH	H-3	C-1, -3, -4, -14, -15, acetate carbonyl
3	5.76 dd (16.0, 10.0)	126.0, CH	H-2, H-4	C-5
4	6.82 d (16.0)	139.0, CH	H-3, H-6, H_3_-16	C-2, -3, -5, -6
5	–	140.4, C	–	–
6	5.44 dq (4.4, 1.6)	118.4, CH	H-4, H-7, H_3_-16	C-4, -8
7	5.10 d (4.4)	76.8, CH	H-6	C-5, -6
8	–	69.9, C	–	–
9	4.36 d (9.6)	74.6, CH	H-10	C-1, -8, -10, -11, -17
10	2.08 d (4.8)	38.5, CH	H-9, H-11	C-1, -2, -8, -9, -11, -14, -15, -20
11	2.23 m	39.2, CH	H-10, H-12, H_3_-20	C-1, -10, -12, -13, -20
12	4.98 m	70.3, CH	H-11, H_2_-13	C-20, -1′
13	2.03 m; 1.84 dt (14.4, 3.2)	26.3, CH_2_	H-12, H-14	C-12
14	4.95 t (3.2)	74.3, CH	H_2_-13	C-15, acetate carbonyl
15	1.43 s	16.1, CH_3_	–	C-1, -2, -10, -14
16	1.89 br s	23.5, CH_3_	H-4, H-6	C-4, -5, -6
17	–	63.4, C	H_3_-18	–
18	1.52 s	10.0, CH_3_	H-17	C-7, -8, -19
19	–	170.7, C	–	–
20	1.15 d (7.2)	10.5, CH_3_	H-11	C-10, -11, -12
OAc-2	–	169.9, C	–	–
	1.98 s	21.2, CH_3_	–	Acetate carbonyl
OAc-14	–	170.4, C	–	–
	2.09 s	21.3, CH_3_	–	Acetate carbonyl
OC(O)Pr-12 1′2′3′4′	–	–	–	–
1′	–	173.0, C	–	–
2′	2.26 t (7.2)	36.3, CH_2_	H_2_-3′	C-1′, -3′, -4′
3′	1.61 sext (7.2)	18.4, CH_2_	H_2_-2′, H_3_-4′	C-1′, -2′, -4′
4′	0.94 t (7.2)	13.7, CH_3_	H_2_-3′	C-2′, -3′

**Table 3:** ^1^H (400 MHz, CDCl_3_) and ^13^C (100 MHz, CDCl_3_) NMR data and ^1^H–^1^H COSY and HMBC correlations for briarane **3**.

Position	δ_H_ (*J* in Hz)	δ_C_, Multiple	^1^H–^1^H COSY	HMBC
1	–	45.7, C	–	–
2	4.72 d (6.0)	73.8, CH	H_2_-3	C-1, -4, -10, -14, -15, acetate carbonyl
3	3.05 m; 1.92 m	40.8, CH_2_	H-2, H-4	C-1, -4, -5
4	4.23 dd (12.4, 5.2)	71.3, CH	H_2_-3	C-5, -6, -16
5	–	147.5, C	–	–
6	5.49 dt (9.6, 1.2)	122.0, CH	H-7, H_3_-16	C-4, -16
7	6.22 d (9.6)	73.4, CH	H-6	C-5, -6
8	–	71.0, C	–	–
9	4.45 dd (6.0, 3.6)	72.2, CH	H-10, OH-9	C-7, -8, -11
10	2.29 d (3.6)	42.5, CH	H-9	C-1, -8, -9, -11, -15
11	–	63.6, C	–	–
12	3.05 d (2.8)	61.4, CH	H_2_-13	n. o. ^a^
13	2.08 m	25.2, CH_2_	H-12, H-14	n. o.
14	4.73 br s	73.8, CH	H_2_-13	C-1, -2, -10, -12, -15, acetate carbonyl
15	1.19 s	16.0, CH_3_	–	C-1, -10, -14
16	2.11 d (1.2)	25.5, CH_3_	H-6	C-4, -5, -6
17	–	62.5, C	–	–
18	1.67 s	9.4, CH_3_	–	C-8, -17, -19
19	–	171.8, C	–	–
20	1.35 s	24.5, CH_3_	–	C-10, -11, -12
OAc-2	–	170.7, C	–	–
	1.98 s	21.1, CH_3_	–	Acetate carbonyl
OAc-14	–	170.6, C	–	–
	2.01 s	21.1, CH_3_	–	Acetate carbonyl
OH-19	2.89 d (6.0)	–	H-9	C-8

^a^ n. o. = not observed.

**Figure 3:**
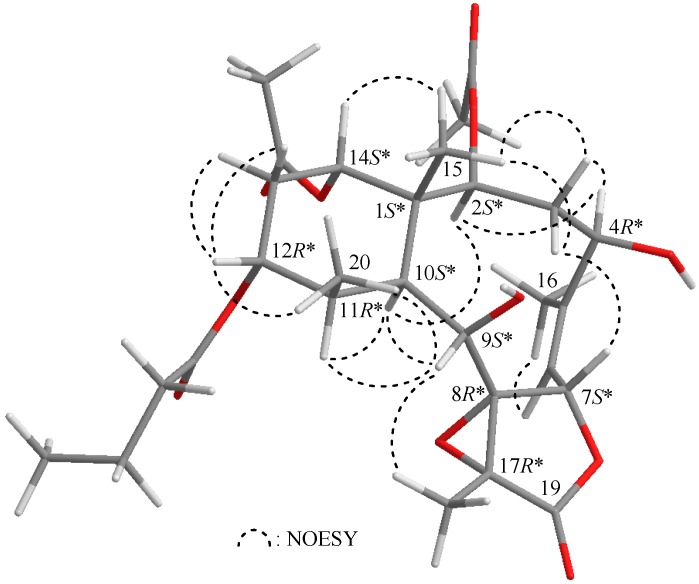
Selected protons with key NOESY correlations of **4**.

Briarenolide ZIV (**4**) was obtained as a white powder, and the molecular formula of **4** was determined to be C_28_H_40_O_11_ (9° of unsaturation) by HRESIMS at *m*/*z* 575.24645 (calcd. for C_28_H_40_O_11_ + Na, 575.24628). The IR spectrum of **4** showed three bands at 3444, 1778 and 1732 cm^−1^, consistent with the presence of hydroxy, γ-lactone and ester carbonyl groups. Carbonyl resonances in the ^13^C NMR spectrum of **4** showed signals at δ_C_ 173.9, 173.2, 170.8 and 170.4, which revealed the presence of a γ-lactone and three esters in **4** (Table 4), of which, two of the esters were identified as acetates based on the presence of two acetyl methyl resonances in the ^1^H NMR spectrum of **4** at δ_H_ 1.97 (2 × 3H, s) (Table 4). The other ester was found to be an *n*-butyrate group based on ^1^H NMR studies, which revealed seven contiguous protons (δ_H_ 0.94, 3H, t, *J* = 7.2 Hz; 1.65, 2H, sextet, *J* = 7.2 Hz; 2.23, 2H, t, *J* = 7.2 Hz). According to the ^1^H and ^13^C NMR spectra, **4** was found to have a γ-lactone moiety (δ_C_ 173.9, C-19) and a trisubstituted olefin (δ_C_ 145.4, C-5; 121.6, CH-6; δ_H_ 5.32, 1H, d, *J* = 8.8 Hz, H-6). The presence of a tetrasubstituted epoxide that contained a methyl substituent was established based on the signals of two oxygenated quaternary carbons at δ_C_ 71.8 (C-8) and 63.7 (C-17) and confirmed by the proton signals of a methyl singlet at δ_H_ 1.51 (3H, s, H_3_-18). Thus, from the NMR data, five degrees of unsaturation were accounted for, and **4** was identified as a tetracyclic compound. From the ^1^H–^1^H COSY spectrum of **4** (Table 4), three different structural units, including C-2/-3/-4, C-6/-7 and C-9/-10/-11/-12/-13/-14, were identified. From these data and the HMBC correlation results (Table 4), the connectivity from C-1 to C-14 could be established. A methyl attached at C-5 was confirmed by an allylic coupling between H_3_-16/H-6 and by the HMBC correlations between H_3_-16/C-4, -5 and -6. The C-15 and C-20 methyl groups were identified as being positioned at C-1 and C-11 from the HMBC correlations between H_3_-15/C-1, -2, -10, -14 and H_3_-20/C-10, -11, -12, respectively. Furthermore, the acetate esters positioned at C-2 and C-14 were established by the HMBC correlations between δ_H_ 4.97 (H-2) and 4.70 (H-14) and the acetate carbonyls at δ_C_ 170.4 and 170.8, respectively. The location of an *n*-butyrate group in **4** was verified by an HMBC correlation between H-12 (δ_H_ 4.83) and the n-butyrate carbonyl carbon (δ_C_ 173.2) (Table 4). These data, together with the HMBC correlations between H_3_-18/C-8, -17 and -19, established the main molecular framework of **4**. The NMR data of **4** were found to be similar to those of a known briarane, excavatolide Z (**9**) [8] (Figure 1), except that the signals corresponding to the 4-hydroxy group in **4** were not present in **9**, and an 11β-hydroxy group was found in **9**. The correlations from NOESY signals of **4** (Figure 3) also showed that the relative configurations of most chiral centers of **4** were similar to those of **9**. H-10 exhibited interactions with H-2 and H-11, and H-2 correlated with H-4, indicating that the hydroxy group at C-4 and the methyl group at C-11 were β-oriented; additionally, briarenolide ZIV (**4**) was found to be the 4β-hydroxy-11-dehydroxy-11β-methyl derivative of **9**.

**Table 4:** ^1^H (400 MHz, CDCl_3_) and ^13^C (100 MHz, CDCl_3_) NMR data and ^1^H–^1^H COSY and HMBC correlations for briarane **4**.

Position	δ_H_ (*J* in Hz)	δ_C_, Multiple	^1^H–^1^H COSY	HMBC
1	–	46.1, C	–	–
2	4.97 d (8.0)	74.9, CH	H_2_-3	C-1, -4, -10, -15, acetate carbonyl
3	3.22 dd (15.2, 12.0); 1.93 m	39.7, CH_2_	H-2, H-4	C-1, -4
4	4.16 dd (12.0, 5.2)	71.3, CH	H_2_-3	C-3, -5, -6, -16
5	–	145.4, C	–	–
6	5.32 d (8.8)	121.6, CH	H-7, H_3_-16	C-4, -16
7	6.14 d (8.8)	75.4, CH	H-6	C-5, -6, -19
8	–	71.8, C	–	–
9	3.79 br s	74.1, CH	H-10	C-1, -7, -8, -10, -11, -17
10	2.39 d (5.2)	37.2, CH	H-9, H-11	C-1, -2, -8, -9, -11, -12, -14, -15, -20
11	1.88 m	43.2, CH	H-10, H-12, H_3_-20	C-1, -10, -12, -20
12	4.83 br s	72.1, CH	H-11, H_2_-13	C-10, -14, -1′
13	2.11 m; 1.95 m	24.6, CH_2_	H-12, H-14	C-11, -12, -14
14	4.70 br s	74.2, CH	H_2_-13	C-1, -2, -10, -12, -15, acetate carbonyl
15	1.32 s	15.2, CH_3_	–	C-1, -2, -10, -14
16	2.05 d (1.2)	25.3, CH_3_	H-6	C-4, -5, -6
17	–	63.7, C	–	–
18	1.51 s	9.7, CH_3_	–	C-8, -17, -19
19	–	173.9, C	–	–
20	1.25 d (7.2)	15.2, CH_3_	H-11	C-10, -11, -12
OAc-12	–	170.4, C	–	–
	1.97 s	21.2, CH_3_	–	Acetate carbonyl
OAc-14	–	170.8, C	–	–
	1.97 s	21.5, CH_3_	–	Acetate carbonyl
OC(O)Pr-12 1′2′3′4′	–	–	–	–
1′	–	173.2, C		–
2′	2.23 t (7.2)	36.6, CH_2_	H_2_-3′	C-1′, -3′, -4′
3′	1.65 sext (7.2)	18.5, CH_2_	H_2_-2′, H_3_-4′	C-1′, -2′, -4′
4′	0.94 t (7.2)	13.6, CH_3_	H_2_-3′	C-2′, -3′

Briarenolide ZV (**5**) was obtained as a white powder and had the molecular formula C_24_H_30_O_10_, as determined by HRESIMS at *m*/*z* 505.20460 (calcd. for C_24_H_30_O_10_ + Na, 505.20442) (10° of unsaturation). The IR spectrum of **5** showed bands at 3445, 1770 and 1732 cm^−1^, consistent with the presence of hydroxy, γ-lactone and ester carbonyl groups. Comparison of the ^1^H and distortioneless enhancement by polar transfer (DEPT) spectra with the molecular formula revealed that there must be three exchangeable protons, requiring the presence of three hydroxy groups. In addition, it was found that the spectral data (IR, ^1^H and ^13^C NMR) of **5** (Table 5) were similar to those of a known briarane, excavatolide Z (**9**) [8] (Figure 1), except that **9** exhibited signals representing an *n*-butyrate substitution, which were replaced by a hydroxy group in **5**. The results of ^1^H–^1^H COSY and HMBC correlations fully supported the positions of functional groups, and hence, briarenolide ZV (**5**) was found to be the 12-*O*-debutyryl derivative of **9**.

The new briarane, briarenolide ZVI (**6**), had a molecular formula of C_26_H_36_O_11_ as determined by HRESIMS at *m*/*z* 547.21473 (calcd. for C_26_H_36_O_11_ + Na, 547.21498). Thus, nine degrees of unsaturation were therefore determined for the molecule of **6**. In addition, the spectral data (IR, ^1^H and ^13^C NMR) (Table 6) of **6** were found to be similar to those of a known briarane, excavatolide E (**10**) [9] (Figure 1). However, the NMR spectra revealed that the signals representing the C-4 methylene group in **10** were replaced by those of an additional acetoxy group. In the NOESY experiment of **6**, H-10 gives correlations to H-2, H-9, H-11 and H-12, but not to H_3_-15 and H_3_-20, and H-2 was found to show a correlation with H-4, indicating that these protons (H-2, H-4, H-9, H-10, H-11 and H-12) are located on the same face of the molecule and assigned as α-protons, since the C-15 and C-20 methyls are the β-substituents at C-1 and C-11, respectively. The signal of H_3_-20 showed a correlation with H_3_-18, indicating that H_3_-18 and 8,17-epoxide group were β- and α-oriented, respectively, in the γ-lactone ring in **6**. H-4 correlated with H-2, but not with H-7 and H_3_-15, indicating that H-7 was β-oriented. H-14 was found to exhibit nuclear Overhauser effect (NOE) responses with H-2 and H_3_-15, but not with H-10, revealing the β-orientation of this proton. Thus, based on the above findings, Compound **6** was found to be the 4β-acetoxy derivative of **10**, with a structure as described by Formula **6**. Furthermore, the chemical shifts for H_3_-18 in briaranes **4**, **5** and **6** were found to appear at δ_H_ 1.51, 1.68 and 1.57, respectively, indicating that the 11β-hydroxy group in **5** led to a downfield chemical shift for H_3_-18.

**Table 5:** ^1^H (400 MHz, CDCl_3_) and ^13^C (100 MHz, CDCl_3_) NMR data and ^1^H–^1^H COSY and HMBC correlations for briarane **5**.

Position	δ_H_ (*J* in Hz)	δ_C_, Multiple	^1^H–^1^H COSY	HMBC
1	–	48.6, C	–	–
2	5.02 d (7.2)	75.7, CH	H_2_-3	C-1, -3, -4, -10, -14, -15, acetate carbonyl
3	2.86 td (15.2, 5.2); 1.59 m	32.5, CH_2_	H-2, H_2_-4	n. o. ^a^
4	2.50 br d (15.2); 1.91 m	28.7, CH_2_	H_2_-3	n. o.
5	–	146.0, C	–	–
6	5.28 d (9.6)	117.9, CH	H-7, H_3_-16	C-4
7	5.50 d (9.6)	75.1, CH	H-6	C-5
8	–	71.1, C	–	–
9	4.65 dd (5.6, 2.0)	69.7, CH	H-10, OH-9	C-7, -8, -10, -11, -17
10	2.13 br s	44.0, CH	H-9	C-9
11	–	78.6, C	–	–
12	3.43 br d (10.0)	76.6, CH	H_2_-13, OH-12	n. o.
13	2.32 m; 1.92 m	26.5, CH_2_	H-12, H-14	n. o.
14	4.99 t (2.8)	77.5, CH	H_2_-13	C-1, -10, -15, acetate carbonyl
15	1.42 s	15.9, CH_3_	–	C-1, -2, -10, -14
16	2.00 s	26.9, CH_3_	H-6	C-4, -5, -6
17	–	63.4, C	–	–
18	1.68 s	9.6, CH_3_	–	C-8, -17, -19
19	–	171.6, C	–	
20	1.41 s	31.1, CH_3_	–	C-10, -11, -12
OAc-2	–	170.8, C	–	–
	1.99 s	21.4, CH_3_	–	Acetate carbonyl
OAc-14	–	169.8, C	–	–
	2.03 s	21.7, CH_3_	–	Acetate carbonyl
OH-9	2.45 br s	–	H-9	n. o.
OH-12	2.74 d (10.0)	–	H-12	n. o.

^a^ n. o. = not observed.

**Figure 4:**
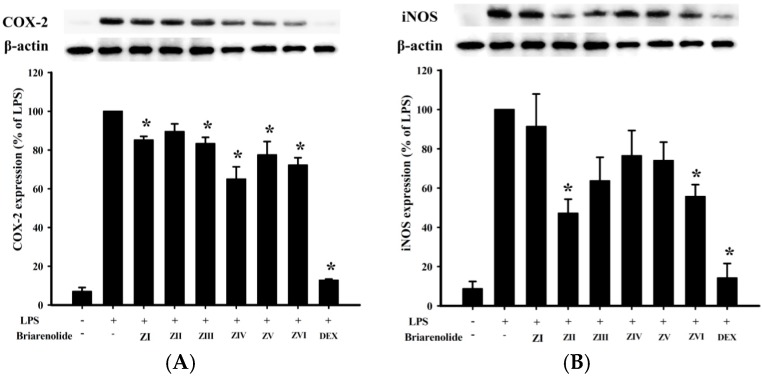
Effects of briarenolides ZI–ZVI (**1**–**6**) on pro-inflammatory cyclooxygenase 2 (COX-2) and inducible nitric oxide synthase (iNOS) protein expressions in lipopolysaccharide (LPS)-stimulated murine macrophage cell line RAW264.7. (**A**) Relative density of the COX-2 Western blot; (**B**) relative density of the iNOS Western blot. The relative intensity of the LPS-stimulated group was taken to be 100%. Band intensities were quantified by densitometry and are indicated as the percentage change relative to that of the LPS-stimulated group. Briarenolides ZII (**2**) and ZVI (**6**) and DEX significantly inhibited LPS-induced iNOS protein expression (<60%) in macrophages. The experiments were repeated three times (* *p* < 0.05, significantly different from the LPS-stimulated group).

In an *in vitro* anti-inflammatory activity assay, Western blot analysis was used to evaluate the upregulation of the pro-inflammatory cyclooxygenase 2 (COX-2) and inducible nitric oxide synthase (iNOS) protein expressions in lipopolysaccharide (LPS)-stimulated RAW264.7 macrophage cells. At a concentration of 10 μM, briarenolides ZII (**2**) and ZVI (**6**) were found to significantly reduce the levels of iNOS to 47.2% and 55.7%, respectively, in comparison to the control cells stimulated with LPS only (Figure 4 and Table 7). By using trypan blue staining, it was observed that briarenolides ZI–ZVI (**1**–**6**) did not induce significant cytotoxicity in RAW264.7 macrophage cells.

**Table 6:** ^1^H (400 MHz, CDCl_3_) and ^13^C (100 MHz, CDCl_3_) NMR data and ^1^H–^1^H COSY and HMBC correlations for briarane **6**.

Position	δ_H_ (*J* in Hz)	δ_C_, Multiple	^1^H–^1^H COSY	HMBC
1	–	46.1, C	–	–
2	4.87 d (8.0)	73.6, CH	H_2_-3	C-1, -3, -4, -10, -14, -15, acetate carbonyl
3	3.16 dd (15.6, 12.8); 1.91 m	37.6, CH_2_	H-2, H-4	C-1, -2, -4, -5
4	5.01 dd (12.8, 5.6)	72.7, CH	H_2_-3	C-3, -5, -6, -16, acetate carbonyl
5	–	144.1, C	–	–
6	5.39 d (9.2)	122.7, CH	H-7, H_3_-16	C-4, -16
7	5.92 d (9.2)	74.5, CH	H-6	C-5, -6, -19
8	–	71.7, C	–	–
9	3.91 br s	74.7, CH	H-10, OH-9	n. o. ^a^
10	2.20 dd (4.8, 2.2)	41.6, CH	H-9, H-11	C-1, -2, -11, -15, -20
11	1.99 m	44.7, CH	H-10, H-12, H_3_-20	n. o.
12	4.04 dt (8.8, 3.6)	67.0, CH	H-11, H_2_-13	n. o.
13	1.84 m	29.0, CH_2_	H-12, H-14	C-1, -12
14	4.78 t (2.8)	76.2, CH	H_2_-13	C-10, -12, acetate carbonyl
15	1.31 s	15.4, CH_3_	–	C-1, -2, -10, -14
16	2.13 s	25.3, CH_3_	H-6	C-4, -5, -6
17	–	63.3, C	–	–
18	1.57 s	10.2, CH_3_	–	C-8, -17, -19
19	–	172.0, C	–	–
20	1.19 d (7.2)	9.5, CH_3_	H-11	C-10, -11, -12
OAc-2	–	170.2, C	–	–
	1.99 s	21.5, CH_3_	–	Acetate carbonyl
OAc-4	–	170.4, C	–	–
	2.01 s	21.0, CH_3_	–	Acetate carbonyl
OAc-14	–	170.5, C	–	–
	1.99 s	21.2, CH_3_	–	Acetate carbonyl
OH-9	2.95 br s	–	H-9	n. o.

^a^ n. o. = not observed.

**Table 7:** The effect of briarenolides ZI–ZVI (**1**–**6**) on LPS-induced COX-2 and iNOS protein expression in macrophage.

Compounds	COX-2	iNOS
	Expression (% of LPS)	Expression (% of LPS)
Control	6.9 ± 2.1	8.7 ± 3.8
LPS	100 ± 0	100 ± 0
ZI (**1**)	85.1 ± 1.9	91.4 ± 16.6
ZII (**2**)	89.5 ± 4.0	47.2 ± 7.2
ZIII (**3**)	83.3 ± 3.3	63.7 ± 12.0
ZIV (**4**)	65.0 ± 6.4	76.4 ± 13.0
ZV (**5**)	77.5 ± 6.9	74.0 ± 9.4
ZVI (**6**)	72.2 ± 3.8	55.7 ± 6.1
DEX ^a^	12.8 ± 0.6	14.2 ± 7.3

^a^ Dexamethasone (DEX) was used as a positive control; COX-2: cyclooxygenase 2; iNOS: inducible nitric oxide synthase; LPS: liposaccharide.

## 3. Experimental Section

### 3.1. General Experimental Procedures

Melting points were determined using a Fargo apparatus (Panchum Scientific Corp. Kaohsiung, Taiwan), and the values were uncorrected. Optical rotation values were measured with a Jasco P-1010 digital polarimeter (Japan Spectroscopic Corporation, Tokyo, Japan). IR spectra were obtained with an FT-IR spectrophotometer (Digilab FTS 1000; Varian Inc., Palo Alto, CA, USA); peaks are reported in cm^−1^. NMR spectra were recorded on a 400-MHz Varian Mercury Plus NMR spectrometer (Varian Inc.) using the residual CHCl_3_ signal (δ_H_ 7.26 ppm) as the internal standard for ^1^H NMR and CDCl_3_ (δ_C_ 77.1 ppm) for ^13^C NMR. Coupling constants (*J*) are given in Hz. ESIMS and HRESIMS were recorded using a Bruker 7 Tesla solariX FTMS system (Bruker, Bremen, Germany). Column chromatography was performed using 230–400 mesh silica gel (Merck, Darmstadt, Germany). TLC was carried out on precoated 0.25 mm-thick Kieselgel 60 F_254_ (Merck); spots were visualized by spraying with 10% H_2_SO_4_ solution followed by heating. Normal-phase HPLC (NP-HPLC) was performed using a system equipped with a Hitachi L-7110 pump (Hitachi Ltd., Tokyo, Japan), a Hitachi L-7455 photodiode array detector and an injection port (7725; Rheodyne LLC, Rohnert Park, CA, USA). A semi-preparative normal-phase LiChrospher column (Hibar 250 mm × 10 mm, Si 60, 5 μm, Merck) was used for HPLC. Reverse-phase HPLC (RP-HPLC) was performed with a system equipped with a Hitachi L-7100 pump, a Hitachi L-2455 photodiode array detector, a Rheodyne 7725 injection port and a 25 cm × 10 mm Polaris 5 C-18-A column (5 μm; Varian Inc., Palo Alto, CA, USA).

### 3.2. Animal Material

Specimens of *Briareum* sp. were collected by hand by scuba divers in an area off the coast of southern Taiwan in July 2011 and stored in a freezer. A voucher specimen was deposited in the National Museum of Marine Biology & Aquarium (NMMBA-TW-SC-2011-77) [10,11,12,13,14].

### 3.3. Extraction and Isolation

Sliced bodies of *Briareum* sp. (wet weight, 6.32 kg; dry weight, 2.78 kg) were extracted with a solvent mixture of methanol (MeOH) and dichloromethane (DCM) (1:1). The extract was partitioned between ethyl acetate (EtOAc) and H_2_O. The EtOAc layer was separated on silica gel followed by elution chromatography with a mixture of *n*-hexane/EtOAc (stepwise, 100:1, pure EtOAc) to yield 26 subfractions, A–Z. Fraction V was chromatographed on silica gel and eluted using a mixture of DCM/EtOAc (stepwise, 20:1, pure EtOAc) to afford 14 subfractions, V1–V14. Fraction V9 was separated by NP-HPLC using a mixture of DCM/EtOAc (1:1) to afford 25 subfractions, V9A–V9Y. Fraction V9J was further repurified by RP-HPLC, using a mixture of MeOH/H_2_O (40:60) as the mobile phase to afford **1** (3.7 mg). Fractions M, N, O and P were combined and further separated on silica gel and eluted using n-hexane/EtOAc (stepwise, 4:1, pure EtOAc) to afford 30 subfractions, M1–M30. Fraction M4 was separated by NP-HPLC, using a mixture of DCM/acetone (40:1) to afford 17 subfractions, M4A–M4Q. Fraction M4B was purified by NP-HPLC, using a mixture of DCM/acetone (100:1) to afford 24 subfractions, M4B1–M4B24. Fraction M4B16 was further separated by RP-HPLC, using a mixture of MeOH/H_2_O (stepwise, 30/70–70/30) to afford **2** (60:40, 1.7 mg). Fraction M12 was chromatographed by silica gel and eluted using a mixture of DCM/MeOH (stepwise, 100:1, pure MeOH) to afford 34 subfractions, M12-1–M12-34. Fraction M12-31 was purified by RP-HPLC, using a mixture of MeOH/H_2_O (60:40) to afford **3** (2.7 mg) and **4** (5.0 mg), respectively. Fraction M18 was repurified by NP-HPLC, using a solvent mixture of DCM/acetone (15:1) to obtain 28 subfractions, M18-1–M18-28. Fraction M18-22 was separated by RP-HPLC, using a solvent mixture of MeOH/H_2_O (1:1) to afford **5** (1.0 mg). Fraction Q was separated on silica gel and eluted using n-hexane/EtOAc (stepwise, 4:1, pure EtOAc) to afford 25 subfractions, Q1–Q25. Fraction Q9 was further separated by reverse-phase C18 column, using a solvent mixture of H_2_O/MeOH (stepwise, 80:20, pure MeOH) to afford 18 subfractions, Q9A–Q9R. Fraction Q9G was separated on RP-HPLC and eluted with MeOH/H_2_O (1:1) as the mobile phase to afford **6** (2.0 mg).

Briarenolide ZI (**1**): white powder; mp 292–293 °C; ${\left[\alpha \right]}_{D}^{25}$ −31 (*c* 0.2, CHCl_3_); IR (neat) ν_max_ 3382, 1769, 1715 cm^−1^; ^1^H (400 MHz, CDCl_3_) and ^13^C (100 MHz, CDCl_3_) NMR data (see Table 1); ESIMS: *m*/*z* 555 [M + Na]^+^, 557 [M + 2 + Na]^+^; HRESIMS: *m*/*z* 555.16025 (calcd. for C_24_H_33_ClO_11_ + Na, 555.16036).

Briarenolide ZII (**2**): white powder; mp 87–88 °C; ${\left[\alpha \right]}_{D}^{25}$ −20 (*c* 0.1, CHCl_3_); IR (neat) ν_max_ 3481, 1781, 1733 cm^−1^; ^1^H (400 MHz, CDCl_3_) and ^13^C (100 MHz, CDCl_3_) NMR data (see Table 2); ESIMS: *m*/*z* 557 [M + Na]^+^; HRESIMS: *m*/*z* 557.23552 (calcd. for C_28_H_38_O_10_ + Na, 557.23572).

Briarenolide ZIII (**3**): white powder; mp 173–174 °C; ${\left[\alpha \right]}_{D}^{25}$ +25 (*c* 0.1, CHCl_3_); IR (neat) ν_max_ 3444, 1779, 1732 cm^−1^; ^1^H (400 MHz, CDCl_3_) and ^13^C (100 MHz, CDCl_3_) NMR data (see Table 3); ESIMS: *m*/*z* 503 [M + Na]^+^; HRESIMS: *m*/*z* 503.18858 (calcd. for C_24_H_32_O_10_ + Na, 503.18877).

Briarenolide ZIV (**4**): white powder; mp 152–153 °C; ${\left[\alpha \right]}_{D}^{25}$ +64 (*c* 0.3, CHCl_3_); IR (neat) ν_max_ 3444, 1778, 1732 cm^−1^; ^1^H (400 MHz, CDCl_3_) and ^13^C (100 MHz, CDCl_3_) NMR data (see Table 4); ESIMS: *m*/*z* 575 [M + Na]^+^; HRESIMS: *m*/*z* 575.24645 (calcd. for C_28_H_40_O_11_ + Na, 575.24628).

Briarenolide ZV (**5**): white powder; mp 192–193 °C; ${\left[\alpha \right]}_{D}^{25}$ +15 (*c* 0.1, CHCl_3_); IR (neat) ν_max_ 3445, 1770, 1732 cm^−1^; ^1^H (400 MHz, CDCl_3_) and ^13^C (100 MHz, CDCl_3_) NMR data (see Table 5); ESIMS: *m*/*z* 505 [M + Na]^+^; HRESIMS: *m*/*z* 505.20460 (calcd. for C_24_H_34_O_10_ + Na, 505.20442).

Briarenolide ZVI (**6**): white powder; mp 173–174 °C; ${\left[\alpha \right]}_{D}^{25}$ +70 (*c* 0.3, CHCl_3_); IR (neat) ν_max_ 3446, 1772, 1734 cm^−1^; ^1^H (400 MHz, CDCl_3_) and ^13^C (100 MHz, CDCl_3_) NMR data (see Table 6); ESIMS: *m*/*z* 547 [M + Na]^+^; HRESIMS: *m*/*z* 547.21473 (calcd. for C_26_H_36_O_11_ + Na, 547.21498).

### 3.4. In Vitro Anti-Inflammatory Assay

The murine macrophage (RAW264.7) cell line was purchased from American Type Culture Collection (ATCC) (Manassas, VA, USA). The *in vitro* anti-inflammatory activities of Compounds **1**−**6** were measured by examining the inhibition of LPS-induced upregulation of pro-inflammatory iNOS and COX-2 protein expressions in the macrophage cell line using Western blotting analysis [15,16,17]. Briefly, an inflammation response in macrophages was induced by incubating cells in medium containing only LPS (10 ng/mL) without compounds for 16 h. For the anti-inflammatory activity assay, Compounds **1**−**6** and dexamethasone (10 μM) were added to the cells 10 min before LPS treatment. After incubation, the cells were lysed for Western blot analysis. The immunoreactivity data were calculated with respect to the average optical density of the corresponding (LPS)-stimulated group. Moreover, the effects of Compounds **1**−**6** on the viability of RAW 264.7 cells were also evaluated by trypan blue staining [16,17]. For statistical analysis, the data were analyzed by one-way analysis of variance (ANOVA), followed by the Student–Newman–Keuls *post hoc* test for multiple comparisons. A significant difference was defined as a *p*-value of <0.05.

## 4. Conclusions

Gorgonian corals belonging to the genus *Briareum* are proven to be rich sources of briarane-type compounds. Briarenolides ZII (**2**) and ZVI (**6**) are potentially anti-inflammatory compounds for future development [18,19]. This interesting species was transplanted to culture tanks located in the NMMBA, for extraction of natural material to establish a stable supply of bioactive substances.
